# Clinical Validity of NETest2.0^®^ in Surveillance of Neuroendocrine Tumor Patients: Evidence from a NET Registry Study (NCT02270567)

**DOI:** 10.3390/cancers18091457

**Published:** 2026-05-01

**Authors:** Anthony Gulati, Diane Reidy, Abdel Halim, Kiarash Mashayekhi, David K. Imagawa, Daniel M. Halperin

**Affiliations:** 1 Department of Medical Oncology, Stamford Health, Stamford, CT 06902, USA; agulati@stamhealth.org; 2 Department of Medical Oncology, Duke Cancer Institute, Durham, NC 27110, USA; diane.reidy-lagunes@duke.edu; 3 Wren Laboratories, Branford, CT 06405, USA; 4 Department of Surgery, University of North Dakota School of Medicine & Health Sciences, Grand Forks, ND 58203, USA; kmashay1@hs.uci.edu; 5 Department of Surgery, University of California, Irvine Medical Center, Irvine, CA 92868, USA; dkimagaw@hs.uci.edu; 6 Department of Hematology and Medical Oncology, Emory University, Atlanta, GA 30322, USA; daniel.mark.halperin@emory.edu

**Keywords:** biomarker, liquid biopsy, mRNA, NETest2.0^®^, neuroendocrine, NET

## Abstract

**Background/Objectives:** The NETest is a blood-based, machine learning-enhanced multigene transcript assay designed to detect and monitor neuroendocrine tumors (NETs). This study evaluated the accuracy of the recently validated NETest2.0^®^ (2025) to (1) detect the presence of disease and (2) assess its utility as a clinically meaningful tool for monitoring NET status across diverse patient cohorts, including post-surgical surveillance, observation (“watch-and-wait”), and treatment settings. **Methods:** This registry study (NCT02270567) evaluated two objectives. For Objective 1, 1290 samples from 886 patients, of which 404 had paired follow-up samples, were analyzed for concordance between NETest2.0^®^ and imaging-detectable disease. For Objective 2, paired blood samples (*n* = 404; median interval 7 months [IQR 4–13.8]) from NET patients across specialized centers were assessed. NETest2.0^®^ scores were correlated with clinically adjudicated disease status using imaging as the comparator. Cohorts included post-surgical residual disease detection (*n* = 71), post-surgical recurrence monitoring (*n* = 44), observation (*n* = 72), and treatment monitoring (*n* = 217; somatostatin analogs, PRRT, and other therapies). Analyses were performed by cohort and in aggregate. **Results:** For Objective 1, NETest2.0^®^ (cut-off ≥ 50) demonstrated an AUC of 0.96, sensitivity of 91.9%, specificity of 94.9%, PPV of 98.4%, NPV of 77.1%, and overall accuracy of 92.5%. Performance was consistent across tumor grades and sites. For Objective 2, 286 patients (70.8%) were stable, and 118 (29.2%) had progression or recurrence. NETest2.0^®^ score changes correlated significantly with outcomes: scores decreased in stable patients (median −14.6%) and increased in progressive disease (median + 15.4%; *p* < 0.0001). Any increase (>0%) in score was associated with progression. Diagnostic performance for detecting progression reached a sensitivity of 78.0%, specificity of 98.3%, PPV of 91.1%, NPV of 90.2%, and accuracy of 83.9%. **Conclusions:** NETest2.0^®^ accurately detects disease and provides a clinically actionable tool for monitoring NETs. Its high specificity and predictive performance support risk-adapted surveillance, potentially reducing unnecessary imaging while identifying early progression across diverse clinical settings.

## Simple Summary

The NETest is a blood-based, machine learning-enhanced multigene transcript assay designed to detect and monitor neuroendocrine tumors (NETs). This registry study (NCT02270567) validated NETest2.0^®^ as a non-invasive molecular assay for postoperative monitoring for minimal residual disease, detection of relapse after surgery, surveillance in watch-and-wait settings, and assessment of treatment effectiveness in NET patients. Diagnostic performance for detecting progression reached a sensitivity of 78.0%, specificity of 98.3%, PPV of 91.1%, NPV of 90.2%, and accuracy of 83.9%. The high specificity and predictive performance of NETest2.0^®^ supports its use as a risk-adapted surveillance tool, potentially reducing unnecessary imaging while identifying early progression across diverse clinical settings.

## 1. Introduction

Neuroendocrine cells, which are distributed throughout the body, play a key role in regulating various physiological processes through hormone release. Neuroendocrine tumors (NETs) originate from these cells and can arise in the gastrointestinal tract, pancreas, lungs, and other tissues [1]. They comprise a heterogeneous disease spectrum with variable presentation and often unpredictable progression [2]. Diagnosing NETs typically involves imaging (CT liver triphasic, MRI, PET) and histopathology. Approximately 40–76% of NETs are metastatic at diagnosis [3]; prognosis varies based on primary site, stage, and grade, with well-differentiated (low-grade/low Ki-67) NETs generally having better outcomes [4].

Despite considerable advances in the diagnosis and management of NET patients, challenges remain, particularly in assessing treatment response and monitoring disease progression [5,6]. While preliminary work identifies the potential of miRNAs as early progression biomarkers, this is limited to tissue evaluations [7]. Circulating free DNA shows some potential value in lung NET [8], particularly in the pre-operative setting, but this requires translational validation. More recently, the adipokine Visfatin has been evaluated but only has modest value as a diagnostic biomarker [9]. Chromogranin A (CgA) is a common circulating biomarker for NETs, but its limited sensitivity and positive predictive value diminish its clinical utility for detecting disease progression. Consequently, there remains no adopted biomarker that reliably predicts postoperative recurrence or progression on treatment.

While imaging remains standard practice, it has limitations in detecting microscopic or minimal residual disease (MRD), identifying early metastasis or recurrence, or accurately evaluating treatment response [10]. Imaging requires in-clinic procedures, and repeated events may increase patients’ cumulative radiation exposure from PET and, to a lesser extent, CT, which is known to be associated with risk for secondary cancers [11]. In addition, such advanced imaging systems may not be equally accessible for rural patients. This issue is especially critical given that postoperative surveillance is often recommended for a decade or longer. Up to 40% of patients considered “cured” eventually experience disease recurrence [12,13].

Most recurrences are detected via imaging with CT, MRI, or somatostatin receptor imaging. Recurrences are typically distant (metastatic disease) and are associated with ~3× increased risk of death [14]. The frequency of surveillance imaging in the USA is highly variable and provider-dependent [13]. In Europe, surveillance imaging is largely guided by established clinical guidelines (e.g., ENETS, ESMO) and tailored to tumor grade, stage, and clinical context. A recent US study identified the importance of updating strategies for surveillance, particularly given the financial burden of frequent imaging, and to optimize the sequencing of treatment to better control disease and prolong survival [14]. In Europe, cost-effectiveness and guideline-based care are important factors. Irrespective of the region, there is an ongoing need to optimize surveillance strategies and treatment sequencing.

NETest2.0^®^ is a novel blood-based machine-learning-enhanced multigene neuroendocrine-specific transcript assay that detects NET gene expression signatures. It functions as a liquid biopsy in vitro diagnostic (IVD), assessing tumor molecular activity through algorithmic analysis of quantitative RT-PCR data derived from circulating mRNA. This blood-based test provides a quantitative measurement of the transcriptomic signature based on a 51-gene panel relevant to NETs, regardless of anatomic origins, in addition to housekeeping genes, and generates a numeric score ranging from 0–100, with a clinically validated cutoff of 50 used to indicate the presence of disease [15]. NETest2.0^®^ is intended for use in patients with well-differentiated neuroendocrine tumors (NETs), including gastroenteropancreatic (GEP-NET), bronchopulmonary (BP-NET), and cancer of unknown primary (CUP), to assist in disease monitoring, detection of recurrence or progression, and evaluation of treatment effectiveness during longitudinal patient management [15].

Unlike CgA, which has limited sensitivity and a poor correlation to disease activity, NETest2.0^®^ measures dynamic transcriptomic changes over time. This enables real-time assessment of tumor activity, treatment response, and progression risk, supporting longitudinal disease monitoring rather than single-timepoint biomarker evaluation.

In this study, our objectives were two-fold: first, to examine the concordance between NETest2.0^®^ scores (positive/negative) and adjudicated disease presence by standard-of-care imaging; and second, to evaluate the performance of NETest2.0^®^ in identifying disease progression in a real-world clinical setting. The overarching goal of this investigation was to assess whether NETest2.0^®^ could function as a clinically and biologically reliable tool to monitor NET status across diverse patient cohorts. Overall, we sought to determine whether the NETest2.0^®^ provides clinically meaningful information that may help to guide patient management and potentially reduce reliance on frequent imaging.

## 2. Materials and Methods

**Figure 1:**
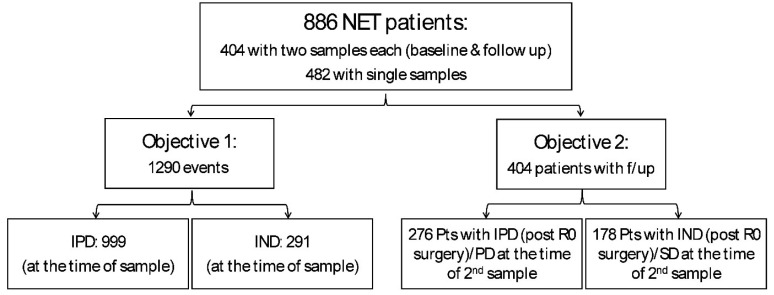
STARD diagram showing an overview of the design, cohort selection, and outcome stratification. Abbreviations: IPD: image-positive disease, IND: image-negative disease; R0: margin-negative curative surgery; PD: progressive disease; SD: stable disease. Responders include stable disease (SD), partial responses (PR), and complete responses (CR). Non-responders are identified as progressive disease (PD). STARD: Standards for Reporting of Diagnostic Accuracy.

Study Design and Patient Cohorts: This registry-based observational study (NCT02270567: RegisterNET) utilized prospectively collected samples and predefined clinical follow-up within routine care pathways to evaluate the clinical utility of NETest2.0^®^ in NET patient surveillance across multiple cohorts representing the diverse management strategies of the disease (Figure 1). Patients were enrolled through a registry-based framework across participating centers and were not hand-selected. Inclusion criteria included a NET diagnosis irrespective of site, grade, or treatment status (GEP-NET, BP-NET) and CUPs. No patient was excluded except if no imaging was available. Enrolment was consecutive where applicable.

Clinical assessments, imaging decisions, and treatment modifications were performed per routine institutional practice, reflecting real-world care pathways. No samples, used in the test or algorithm development, including training and testing sets [15], were included in the current study. Eight hundred and eighty-six subjects were included (Figure 1), comprising 404 individuals with paired samples and 482 with a single sample, yielding a total of 1290 NETest2.0^®^ score measurements.

For Objective 1, NETest2.0^®^ scores were evaluated for concordance with imaging-based detection of disease. Positive and negative NETest2.0^®^ results were compared with findings from CT, MRI, or ^68^Ga-PET-SSA-PET-CT to determine alignment between the assay and macroscopic disease status. The 1290 subjects included 1154 GEP, 75 BP, and 61 CUP. The 1154 GEP cohort included patients with NET of the pancreas (579), small bowel (465), gastric (28), rectum (34), appendix (14), colon (3), esophagus (6), and duodenum (25).

**Figure 2:**
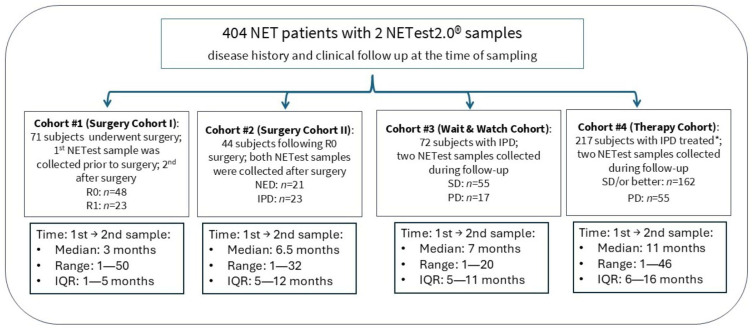
STARD diagram showing an overview of the design, cohort selection, and outcome stratification for Objective 2. Abbreviations: IPD: image-positive disease, IND: image-negative disease; R0: margin-negative curative surgery; PD: progressive disease; SD: stable disease. Responders include stable disease (SD), partial responses (PR), and complete responses (CR). Non-responders are identified as progressive disease (PD). IQR = interquartile range.

For Objective 2, relative changes in NETest2.0^®^ scores between two timepoints for each patient were analyzed alongside clinical follow-up and imaging to assess the NETest2.0^®^’s ability to monitor disease-free status post-curative surgery, disease stability, progression, or recurrence. The 404 patients included 369 with GEP-NETs, 18 with BP-NET, and 17 with CUP. The GEP cohort included patients with cancer of the pancreas (183), small bowel (168), appendix (2), duodenum (3), esophagus (1), gastric (4), and rectum (8). Analyses were performed both for the entire cohort (*n* = 404) and within predefined subgroups. Patients were grouped into four cohorts (Figure 2) based on treatment status and timing of sample collection relative to surgery or therapy. This allowed us to investigate the value of the test in different clinical scenarios.

Comparator Imaging and Clinical Assessment: Imaging comparators were performed as part of routine institutional clinical care and were not study-directed. Modalities included contrast-enhanced CT, MRI, or somatostatin receptor imaging (^68^Ga-SSA PET/CT), selected according to physician judgment and institutional practice guidelines. Importantly, imaging modality selection was independent of NETest2.0^®^ results.

Whenever clinically feasible, the same imaging modality was used for longitudinal comparison within individual patients. Imaging interpretation and clinical response assessments were performed by treating neuroendocrine tumor specialists according to standard radiologic criteria, including RECIST 1.1, where applicable. In this study, 1126 CT/MRI and 128 ^68^Ga-PET were performed.

Objective 1 was set to validate the NETest2.0 as a reliable tool to detect NET, while Objective 2 was set to assess NETes2.0 as a surveillance biomarker to detect minimal residual disease (MRD) in R0 surgery (total resection), tumor relapse, or progression.

Surgical Cohorts: Cohort 1 (*n* = 71; Table 1) was included to address the MRD intention by analyzing NETest2.0 before and a median of three months after surgery, and NETest2.0^®^ scores were correlated with image-assessed evidence of disease. Cohort II (*n* = 44) was included to determine whether NETest2.0^®^ could be used to monitor for recurrence following curative-intent surgery. Two NETest2.0 samples were collected approximately six months apart after surgery to assess for evidence of disease recurrence. Follow-up was conducted according to standard U.S. surgical management guidelines, which included CT/MRI at 3–6, 12, and 18 months [16,17]. Additional imaging modalities, such as ^68^Ga-SSR-PET/CT, were performed at the discretion of the multidisciplinary NET teams.

Active (image-positive) Disease Cohort: Patients with metastatic or unresectable disease were evaluated in two cohorts.

Observation Cohort (Cohort III: *n* = 72; Table 1), patients were evaluated at two intervals, approximately seven months apart, for evidence of disease progression. Imaging was performed at the second timepoint, and disease status—classified as stable or progressive—was evaluated as described. The objective was to determine whether NETest2.0^®^ could be used for monitoring disease progression.

Treatment Cohort (Cohort IV: *n* = 217; Table 1), patients were evaluated at two intervals (~7 months apart) for evidence of treatment response. Patients were receiving systemic therapy, including somatostatin analogs (SSAs; *n* = 48), peptide receptor radionuclide therapy (PRRT: *n* = 152), and a regimen consisting of capecitabine and temozolomide (CAPTEM) (*n* = 5), everolimus (*n* = 5), or chemotherapy (*n* = 7).

Clinical records were reviewed to assess whether changes in NETest2.0^®^ scores correlated with standardized imaging endpoints. Imaging comparators (CT, MRI, or ^68^Ga-SSA PET/CT) were performed per routine institutional practice. Comparator modality was not selected based on NETest results. For each patient, the same imaging modality was used at both timepoints whenever clinically feasible. Specifically, disease status was categorized as stable or progressive. All imaging was adjudicated by treating NET specialists. Disease was considered stable (stable disease [SD], partial responders [PR], or complete responders [CR]) if there was no radiological or clinical evidence of progression. Disease was classified as progressive (PD) if serial imaging met RECIST 1.1 criteria for progression [18], or if there were significant clinical changes indicative of disease progression as assessed by an institution’s standard practice.

Ethics approvals: Ethics approvals were obtained from the Western Institutional Review/WCG Board (WIRB20191743—NET Registry: RegisterNET—A Registry of Neuroendocrine Tumors in the USA (www.clinicaltrials.gov #NCT02270567)) [19]. Blood samples and clinical histories were collected from participants, with all samples and associated health information de-identified in accordance with privacy regulations. Demographic data, clinical status, ongoing treatment, and imaging data were compiled into a centralized database for subsequent analysis.

Blood samples: Peripheral whole blood samples (2 mL) were collected into tubes containing 4 mL of Wren’s proprietary RNA stabilization buffer based on guanidinium hydrochloride as previously described [20]. Since mRNA is extracted from whole blood, no pre-analytical processing was needed. Sampling intervals and clinical assessments followed standard institutional surveillance protocols defined prior to analysis. Blood samples were collected at the time of imaging.

Sample processing: RNA isolation, processing, cDNA synthesis, and qPCR were undertaken as published [15,21,22]. The qPCR data were processed through the NETest2.0^®^ algorithm [15]. Results were expressed as a risk stratification score (0–100) for NET, which was converted into a binary readout (positive/ negative). A clinically standardized cutoff score ≥ 50 [15] was used to detect a neuroendocrine tumor (score ≥ 50 = positive; score < 50 = negative). For patient surveillance, the percent change in a follow-up sample score from the baseline (initial) sample score was calculated. The assay has been analytically validated for its intended use and has been approved by the New York State Department of Health-Clinical Laboratory Evaluation Program (CLEP).

Statistical analysis: Data are presented as positive/negative, median scores (IQR), and mean ± SD of the 0–100 score. For Objective 1, 2 × 2 tables were constructed and AUROC analyses performed, as well as diagnostic metrics, to identify the accuracy of NETest2.0^®^ for detecting disease (*n* = 1290). The accuracy for NETest2.0^®^ in different grades (G1: *n* = 582, G2: *n* = 591, and G3: *n* = 101) was evaluated in a sub-analysis. Concordance analysis was assessed using % overall agreement. A sub-analysis was also performed on the 886 individual patient samples to ensure no bias had been introduced by using paired samples from the 404 subjects.

**Figure 3:**
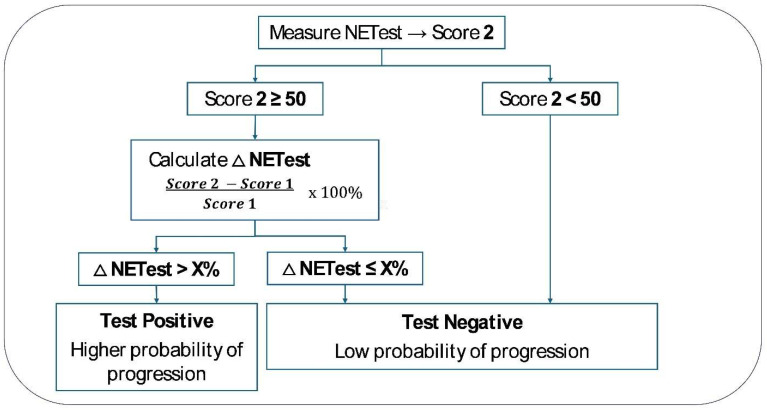
Paradigm used to evaluate changes in NETest score for outcome. This approach is based on the FDA-approved approach for Chromogranin A per the CASPAR study [23]. We evaluated the following Δ thresholds (change between second and first score): >0%, >+5%, >+10%, >+15%, >+20%.

For Objective 2, changes in NETest2.0^®^ score were calculated following the approach used for CgA as a monitor in the CASPAR study [23]. This approach is described in Figure 3.

We evaluated the following Δ for NETest2.0^®^: >0%, +5%, +10%, +15%, +20%. We examined the concordance between progressive disease and those with Δ > threshold compared to those with a negative score and/or Δ < threshold. AUROC analyses and diagnostic metrics were calculated. We examined values both in the entire cohort (*n* = 404) as well as in each of the four sub-cohorts. A sub-analysis was undertaken comparing SSA and PRRT.

The 95% confidence intervals were included for AUCs and diagnostic metrics. These included sensitivity, specificity, positive likelihood ratio (PLR), negative likelihood ratio (NLR), positive predictive value (PPV), negative predictive value (NPV), and overall accuracy.

All analyses were performed using Prism version 9.4 for Windows (GraphPad Software Inc., La Jolla, CA, USA, www.graphpad.com) and MedCalc Statistical Software v23.2.1 (MedCalc Software Ltd., Ostend, Belgium; http://www.medcalc.org; 2017). Statistical significance was defined as *p* < 0.05.

## 3. Results

**Figure 4:**
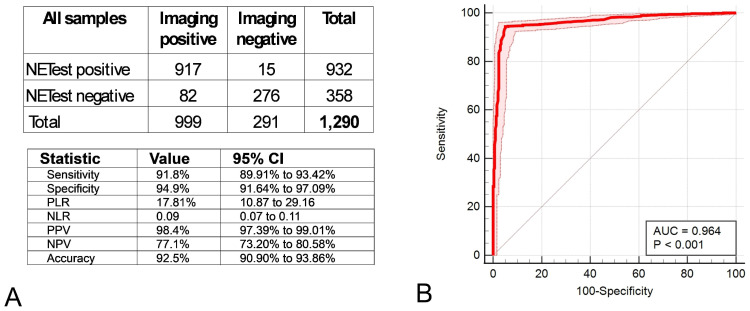
Assessment of NETest2.0^®^ (positive/negative) for detecting NET disease. (**A**). A 2 × 2 table and the corresponding diagnostic metrics for detecting disease. (**B**). AUROC for disease detection. including the Z-statistics (80.64) and significance (*p* < 0.0001). Abbreviations: AUROC: Area Under the Receiver Operator Curve, AUC: Area Under the Curve; NLR = negative likelihood ratio, NPV = negative predictive value, PLR = positive likelihood ratio, PPV = positive predictive value. NLR = negative likelihood ratio, NPV = negative predictive value, PLR = positive likelihood ratio, PPV = positive predictive value.

Objective 1: Evaluation of NETest2.0^®^ score (positive/negative) using imaging as a comparator: Imaging identified detectable disease in 999 cases (77.4%); among those, NETest2.0^®^ was positive in 917 (91.8%) of these cases. In image-negative cases, NETest2.0^®^ was positive in 15/291 (5.2%). The associated metrics are included in Figure 4A (overall accuracy 92.5%). The AUC was 0.96 ± 0.012 (Figure 4B). The overall concordance was 92.48%.

Since imaging was used as the clinical truth in assessing the clinical validity of NETEst2.0^®^, the 1290 samples were considered as events similar to what FDA has considered in the clearance of B·R·A·H·M·S CgA II KRYPTOR assay (510(k) Premarket Notification: B·R·A·H·M·S CgA II KRYPTOR (K222251)) [23]. However, to rule out any bias that might have been introduced by including two samples from the subset of 404 patients with paired samples, a sub-analysis of the 886 patients (baseline samples) was undertaken. Out of the 886 subjects, imaging identified detectable disease in 721 cases (81.4%). In this cohort, Supplementary Figure S1 shows an AUC of 0.92 ± 0.013 and an overall accuracy of 92.9%, which are comparable with the results from the 1290 samples. A sub-analysis restricted to independent baseline samples (*n* = 886) yielded comparable performance, supporting the robustness of the findings.

**Figure 5:**
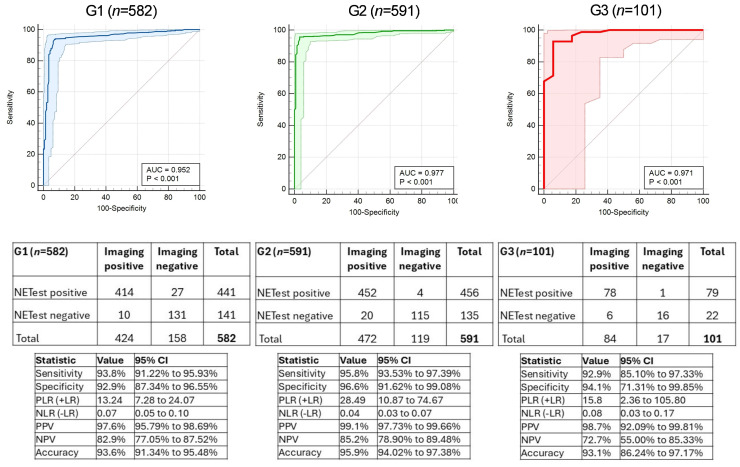
Assessment of NETest (positive/negative) for detecting NET disease based on grade. AUCs for each of the three grades, G1, G2, and G3 tumors. The 2 × 2 tables and the corresponding diagnostic metrics for detecting disease are also included for each of the three grade groups. Abbreviations: AUROC: Area Under the Receiver Operator Curve, AUC: Area Under the Curve; NLR = negative likelihood ratio, NPV = negative predictive value, PLR = positive likelihood ratio, PPV = positive predictive value. NLR = negative likelihood ratio, NPV = negative predictive value, PLR = positive likelihood ratio, PPV = positive predictive value.

Impact of grade on NETest2.0^®^ accuracy for disease detection: Tumor grade was available in 1274 cases (98.9%). AUCs for each of the three grade types ranged from 0.952 ± 0.03 in G1 to 0.98 ± 0.02 in G2 and 0.97 ± 0.02 in G3 (Figure 5). The diagnostic metrics for the NETest2.0^®^ by grade are included. Sensitivities ranged 92.9–95.8%, specificities were 92.9–96.6%, and accuracies were 93.1–95.9%. The overall concordances were 93.46% (G1), 95.9% (G2), and 93.07% (G3).

**Figure 6:**
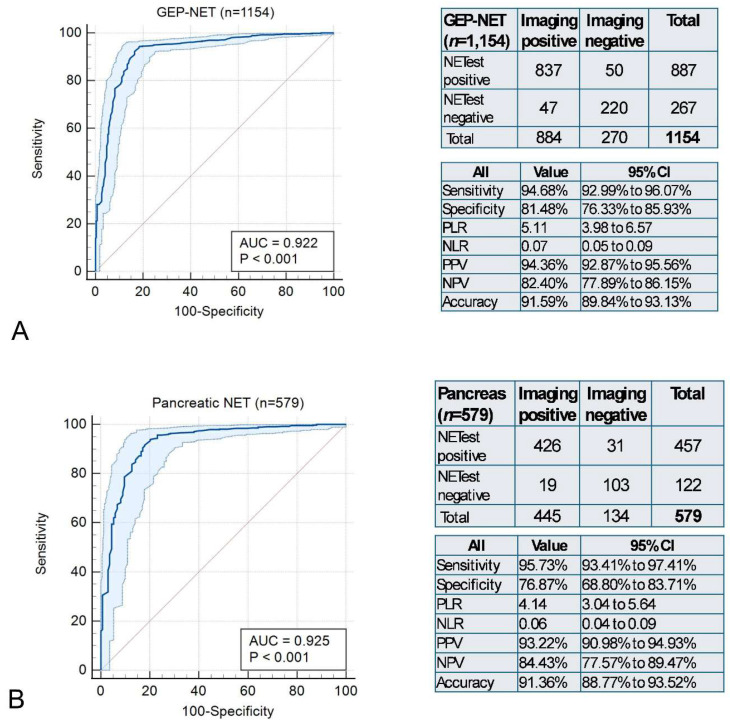

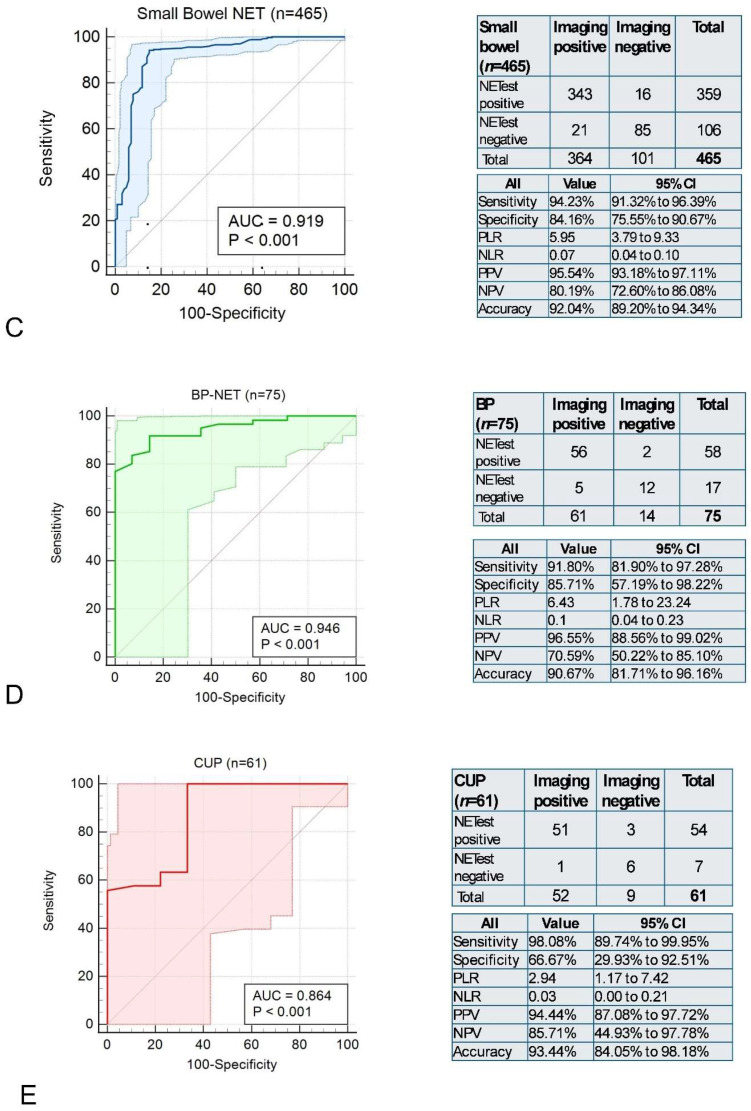
NETest2.0^®^ (positive/negative) for detecting NET disease based on tumor group. (**A**). AUROC for disease detection in 1154 GEP-NETs and the corresponding 2 × 2 tables and diagnostic metrics. (**B**). AUC for pancreatic (*n* = 579) and the corresponding 2 × 2 tables and diagnostic metrics. (**C**). AUC for small bowel NETs (*n* = 465) and the corresponding 2 × 2 tables and diagnostic metrics. (**D**). AUC for bronchopulmonary NETs (*n* = 75) and the corresponding 2 × 2 tables and diagnostic metrics. (**E**). AUC for CUPs (*n* = 61) and the corresponding 2 × 2 tables and diagnostic metrics. Abbreviations: AUROC: Area Under the Receiver Operator Curve, AUC: Area Under the Curve; NLR = negative likelihood ratio, NPV = negative predictive value, PLR = positive likelihood ratio, PPV = positive predictive value.

Impact of tumor site on NETest2.0^®^ accuracy for disease detection: A total of 1154 of the 1290 cases were GEP-NETs. The AUC for detection was 0.92 ± 0.01 (Figure 6A). The AUCs in the pancreatic NET cohort (*n* = 579) were 0.93 ± 0.01 (Figure 6B) vs. 0.92 ± 0.02 in small bowel NETs (*n* = 465) (Figure 6C). BP-NETs (*n* = 75) exhibited a similar AUC (0.95 ± 0.03, Figure 6D) while CUPs (*n* = 61) were associated with an AUC of 0.87 ± 0.07 (Figure 6E). There were no statistically significant differences in detection between any of these tumor groups or types. Overall concordance was high irrespective of tumor location (GEP-NETs: 91.59%, pancreatic NET: 91.36%, small bowel NET: 92.04%, BP-NETs: 90.67%, and CUPs: 93.44%. This subgroup analysis demonstrated consistent performance of NETest2.0^®^ across primary sites.

**Figure 7:**
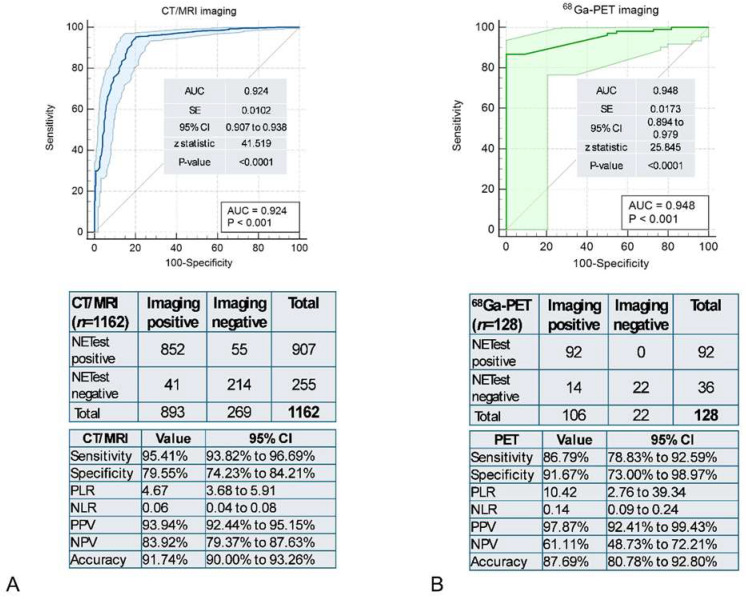
Concordance of NETest2.0^®^ (positive/negative) for detecting NET disease based on anatomical (CT/MRI) versus functional (^68^Ga-PET) imaging. (**A**). AUROC for disease detection using anatomical imaging (*n* = 1126), the corresponding 2 × 2 table, and the diagnostic metrics. (**B**). AUROC for disease detection using functional imaging (*n* = 128), the corresponding 2 × 2 table, and the diagnostic metrics. Abbreviations: AUROC: Area Under the Receiver Operator Curve, AUC: Area Under the Curve; NLR = negative likelihood ratio, NPV = negative predictive value, PLR = positive likelihood ratio, PPV = positive predictive value. NLR = negative likelihood ratio, NPV = negative predictive value, PLR = positive likelihood ratio, PPV = positive predictive value.

NETest2.0^®^ accuracy for disease detection as adjudicated by different imaging modalities: A total of 1162 cases were adjudicated by anatomical (CT/MRI) and 128 by functional (^68^Ga-PET-SSA) imaging. The AUC for anatomical imaging was 0.92 ± 0.01 vs. 0.95 ± 0.02 for functional imaging (Figure 7). There was no significant difference between the two imaging approaches (z-statistic: −1.195, *p* = 0.232). NETest2.0^®^ performance was comparable across imaging modalities. CT/MRI-associated cases demonstrated higher sensitivity (95.4%), whereas PET-associated cases demonstrated higher specificity (91.7%), indicating complementary diagnostic characteristics. Concordance analysis identified 91.74% for CT/MRI and 89.06% for ^68^Ga-PET imaging.

Objective 2: Evaluate changes in NETest2.0^®^ score for monitoring during patient follow-up: To assess the clinical utility of serial NETest2.0^®^ measurements in disease surveillance, changes in paired test results were analyzed across the full cohort (*n* = 404). Patient cohorts are included in Table 1, and details are included in Figure 2.

Patients were stratified by clinical status at the follow-up sampling into two groups:

- NED/SD/PR/CR (*n* = 286): No Evidence of Disease (NED), Stable Disease (SD), or Response (Partial Response [PR]; Complete Response [CR]) to therapy.
- Residual Disease (RD) or Progressive Disease (*n* = 118).

The median interval between paired NETest2.0^®^ samples was 7.0 months (IQR: 4–13.8 months).

**Figure 8:**
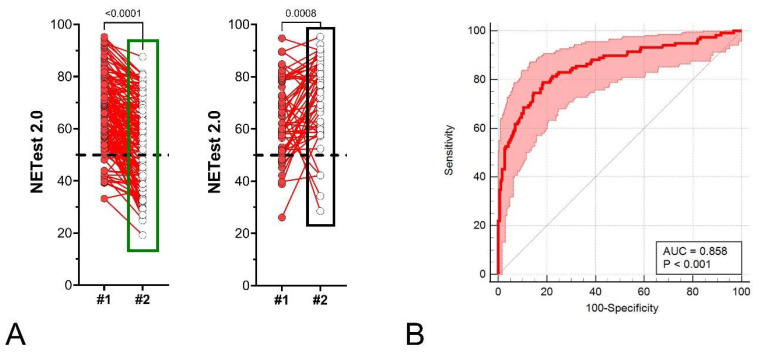
Changes in NETest2.0^®^ score and AUROC analysis (all subjects: *n* = 404). (**A**). Paired NETest2.0^®^ scores in NED/SD/R (**left**) and those who recurred or progressed (**right**). (**B**). AUROC curve for the % change in NETest2.0^®^ (between first and second score) based on the algorithm. The AUC is 0.86 ± 0.023. The 95%CI is 0.82–0.89. The Z-statistic is 15.70 (*p* < 0.0001), the Youden Index is 0.603, and the associated criteria are change > 0% (sensitivity: 78.8%, specificity 81.5%). The green box (**left** panel) highlights the distribution and range of NETest2.0® scores in the NED/SD/responder group, while the black box (**right** panel) highlights the corresponding distribution in the progression/recurrence group, illustrating the shift toward higher scores in progressing patients. The dashed black line is the positive score cut-off of 50 (<50 = negative, ≥50 = positive).

NETest2.0^®^ score changes showed a strong correlation with imaging (Figure 8A).

In the NED/SD/PR/CR group, test values decreased by a median of –14.6% (IQR: –28.2% to −2.5%), indicating a significant relationship (*p* < 0.0001) between score decreases and treatment efficacy or disease stability. In contrast, RD/PD patients demonstrated a median increase of +15.4% (IQR: +1.4% to +35.7%), consistent with disease activity or progression. This was a significant increase (*p* < 0.001). An AUROC analysis identified an AUC of 0.86 ± 0.023 for changes in scores and outcome (Figure 8B). NETest2.0^®^ performance metrics are included in Table 2. The corresponding 2 × 2 tables for each threshold are included in Supplementary Figure S2.

Evaluate NETest2.0^®^ changes in tumor groups: The 404 cases included 386 GEP-NETs, 18 BP-NETs, and 17 CUPs. We evaluated score changes in the 386 GEP-NET cohort (all subjects excluding the 18 with BP-NET) and in the 369 GEP-NET (excluding BP and CUP-NET). Metrics included in Table 3 and Table 4 demonstrated consistent performance across all NETs (*n* = 404), GEP-NETs with CUP (*n* = 386), and GEP-NETs alone (*n* = 369), indicating that classifier performance was not driven by the inclusion of bronchopulmonary or CUP cases. The 2 × 2 tables for each threshold are included in Supplementary Figures S3 and S4.

Evaluate NETest2.0^®^ changes based on disease burden: The 404 cases included 94 localized NETs, 108 NETs with lymph node metastases, and 202 subjects with distant disease (liver disease). We examined score changes in these three cohorts to evaluate the impact of metastatic sites and burden. AUCs for score changes are included in Supplementary Figure S5A–C. These ranged from 0.83 ± 0.048 (localized) to 0.94 ± 0.02 (lymph node) to 0.85 ± 0.032 (liver metastases). Percentage changes in scores are included in Supplementary Figure S5D–F. Those who progressed or exhibited disease recurrence all exhibited increases in score, irrespective of disease burden. These differences were significant (*p* < 0.0001).

Relationship between score changes and follow-up: As changes in NETest2.0^®^ score may depend on the type of intervention (e.g., surgery) or therapy (e.g., PRRT versus SSA), or even whether the patient is being followed up in a watch-and-wait protocol, we next examined the information associated with score changes and these follow-ups.

**Figure 9:**
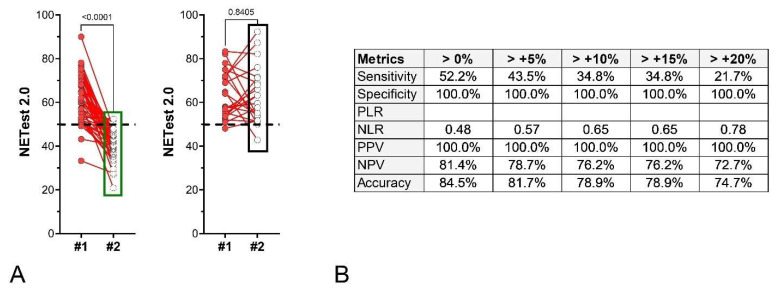
Paired NETest2.0^®^ scores and % change of the second sample score from the initial sample score in surgical subjects (Surgery Cohort I, *n* = 71). (**A**). Paired NETest2.0^®^ scores in the NED group (*n* = 48, **left**) and in the RD group (*n* = 238, **right**). *p*-values = Wilcoxon signed rank test (matched pairs). (**B**). Metrics for score changes (using different thresholds). A change of >0% was associated with the most consistent metrics. Abbreviations: PLR = positive likelihood ratio; NLR = negative likelihood ratio; NPV = negative predictive value. PPV = positive predictive value. The green box (**left** panel) highlights the distribution and range of NETest2.0® scores in the NED/SD/responder group, while the black box (**right** panel) highlights the corresponding distribution in the progression/recurrence group, illustrating the shift toward higher scores in progressing patients. The dashed black line is the score cut-off of 50 (<50 = negative, ≥50 = positive).

Surveillance Cohort I: Seventy-one subjects underwent surgery, with baseline NETest2.0^®^ samples collected preoperatively and follow-up samples a median of three months post-surgery. Forty-eight patients had R0 resections (negative margins, NED) and 23 had R1 resections (microscopic disease in margins). In those with no residual disease, scores significantly (*p* < 0.0001) decreased (62.6 ± 10.8 to 39.7 ± 7.4; Figure 9A—left). Median score changes were –34.7% (IQR: –47.0 to –24.1%). Two of the forty-eight (4.2%) NED were NETest2.0^®^-positive. No significant differences in scores (*p* = 0.84) were noted before surgery (63.4 ± 10.8) and after (63.0 ± 12.7) in those individuals with residual disease (Figure 9A—right). Twenty-two (95.6%) were NETest2.0^®^-positive post-surgery. The metrics associated with different NETest2.0^®^ thresholds (>0–20% changes) are included in Figure 9B. A value of >0% change exhibited the best accuracy metrics.

**Figure 10:**
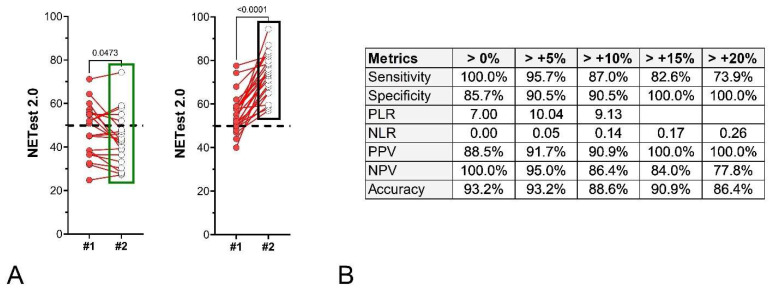
Paired NETest2.0^®^ scores and % change of the second sample score from the initial sample score in surgical subjects (Surgery Cohort II, *n* = 44). (**A**). Paired NETest2.0^®^ scores in the NED group (*n* = 21, **left**) and in the RD group (*n* = 23, **right**). (**B**). Metrics for score changes (using different thresholds). A change of >0% was associated with the most consistent metrics. *p*-values = Wilcoxon signed rank test (matched pairs). The green box (**left** panel) highlights the distribution and range of NETest2.0® scores in the NED/SD/responder group, while the black box (**right** panel) highlights the corresponding distribution in the progression/recurrence group, illustrating the shift toward higher scores in progressing patients. The dashed black line is the score cut-off of 50 (<50 = negative, ≥50 = positive).

Surveillance Cohort II: Forty-four subjects underwent curative surgery, with baseline NETest2.0^®^ samples collected post-operatively (~3 months post-surgery) with follow-up samples at a median of ~6.5 months thereafter. Twenty-one remained disease-free (NED), and 23 exhibited recurrences (image-positive) at follow-up sampling (median: 6.5 months). Median NETest2.0^®^ changes were –6.7% (IQR: −20.5 to 3.2%) in SD (Figure 10A—left) and +29.3% (IQR: 20.4% to 43.1%) in recurrent patients. In those with recurrent disease, scores significantly (*p* < 0.0001) increased (55.2 ± 9.0 to 73.4 ± 9.8; Figure 10A—right). All patients who recurred were NETest2.0^®^-positive. The metrics associated with different NETest2.0^®^ thresholds (>0–20% changes) are included in Figure 10B. A value of >5% change exhibited the best metrics.

**Figure 11:**
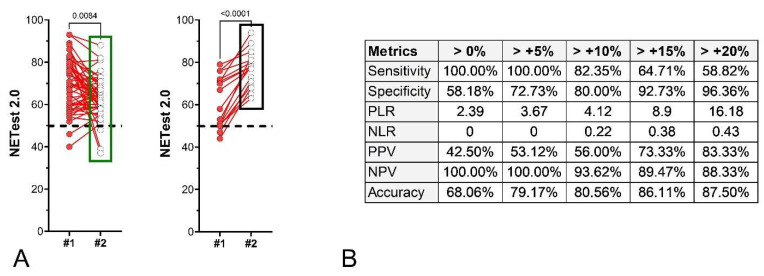
Paired NETest2.0^®^ scores and percent change in the Watch-and-Wait Cohort. (**A**). Paired NETest2.0^®^ scores in the SD group (*n* = 55, **left**) and in the PD group (*n* = 17, **right**). (**B**). Metrics for score changes (using different thresholds). A change of >0% was associated with the most consistent metrics. % Change = Mean ± SD. *p*-values = Wilcoxon signed rank test (matched pairs). SD = stable disease, PD = progressive disease. The green box (**left** panel) highlights the distribution and range of NETest2.0® scores in the NED/SD/responder group, while the black box (**right** panel) highlights the corresponding distribution in the progression/recurrence group, illustrating the shift toward higher scores in progressing patients. The dashed black line is the score cut-off of 50 (<50 = negative, ≥50 = positive).

Watch-and-Wait Cohort: Seventy-two patients with image-positive disease undergoing surveillance without active treatment had paired NETest2.0^®^ samples. Fifty-five had stable disease (SD), and 17 showed progression (PD) at follow-up. Median NETest2.0^®^ changes were −5.0% in SD and +27.0% in PD patients. Changes in scores were significantly different between the two outcomes (Figure 11A). For those with stable disease, scores were initially 69.3 ± 12.2, with a follow-up of 63.7 ± 9.6 (*p* = 0.0084). For those who developed progression, initial scores were 61.1 ± 12.0 with a follow-up of 77.2 ± 8.3 (*p* < 0.0001). The metrics associated with different NETest2.0^®^ thresholds (>0–20% changes) are included in Figure 11B. A value of >+20% change exhibited the highest metrics for progression.

**Figure 12:**
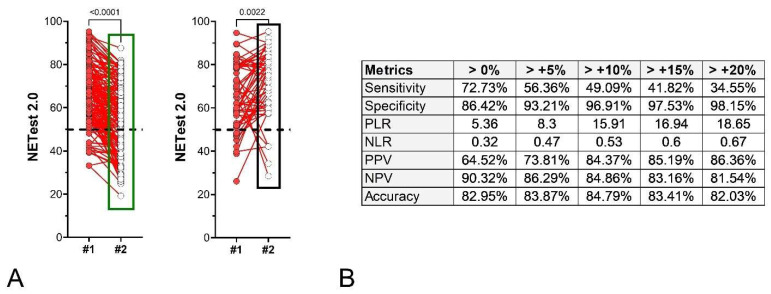
Paired NETest2.0^®^ scores and % change of the second sample score from the initial sample score in treated subjects (Treatment Cohort, *n* = 217). (**A**). Paired NETest2.0^®^ scores in the SD group (*n* = 152, **left**) and in the PD group (*n* = 55, **right**). (**B**). Metrics for score changes (using different thresholds). A change of >0% was associated with the most consistent metrics. *p*-values = Wilcoxon signed rank test (matched pairs). SD = stable disease, PD = progressive disease. The green box (**left** panel) highlights the distribution and range of NETest2.0^®^ scores in the NED/SD/responder group, while the black box (**right** panel) highlights the corresponding distribution in the progression/recurrence group, illustrating the shift toward higher scores in progressing patients. The dashed black line is the score cut-off of 50 (<50 = negative, ≥50 = positive).

**Treatment Cohort:** Two hundred and seventeen patients with image-positive disease receiving active treatment had paired NETest2.0^®^ samples. Treatments included SSA (*n* = 48), PRRT (*n* = 152), CAPTEM (*n* = 5), Everolimus (*n* = 5), and chemotherapy (*n* = 7). One hundred and fifty-two patients were considered responders (R, including PR + CR + SD), and fifty-five were progressive (PD). Median NETest2.0^®^ score decreased by –13.4% (IQR: −27.4 to −3.6%) in those who were responding and increased by +10.2% (IQR: −0.9 to +35.0%) in progressors. Changes in scores were significantly different (*p* < 0.0001) between each of the two outcomes (Figure 12A). For those with stable disease, scores were initially 66.0 ± 14.1, with a follow-up of 55.3 ± 15.9 (*p* = 0.0084). For those who developed progression, initial scores were 65.0 ± 15.0 with the follow-up of 71.7 ± 15.3 (*p* < 0.0001). The metrics associated with different NETest2.0^®^ thresholds (>0–20% changes) are included in Figure 12B.

**Figure 13:**
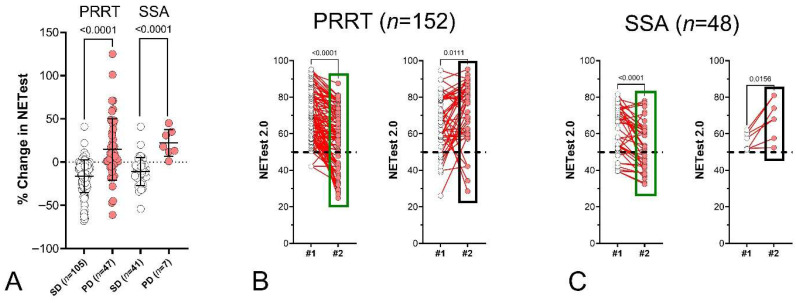
Paired NETest 2.0 scores and percent change in the SSA and PPRT treated individuals: Sub-analysis of changes in pancreatic and small bowel NETs. (**A**). % changes in SD vs. PD for both PRRT and SSA-treated cohorts. Changes were significantly different between responders and non-responders, irrespective of the treatment type. (**B**). Paired NETest 2.0 scores in responders/SD (**left**) and those who progressed (**right**) on PRRT. Scores decreased in responders and significantly increased in progressors. (**C**). Paired NETest 2.0 scores in responders/SD (**left**) and those who progressed (**right**) on SSAs. Scores decreased in responders and significantly increased in progressors. *% Change = Mean ± SD.* SD = stable disease, PD = progressive disease. The green box (**left** panel) highlights the distribution and range of NETest2.0^®^ scores in the NED/SD/responder group, while the black box (**right** panel) highlights the corresponding distribution in the progression/recurrence group, illustrating the shift toward higher scores in progressing patients. The dashed black line is the score cut-off of 50% (<50 = negative, ≥50 = positive).

A sub-analysis identified that NETest2.0^®^ performance was consistent between PRRT and SSA subgroups (Figure 13A–C), with increases in NETest2.0^®^ scores identifying all non-responders and decreases correlating with treatment response.

## 4. Discussion

This study demonstrates that NETest2.0^®^ accurately detects NET disease and that serial NETest2.0^®^ measurements could serve as a clinically and biologically reliable tool to monitor NET status across diverse patient cohorts. For disease detection, a positive NETest2.0^®^ score (≥50) exhibited an AUC of 0.96 and an overall accuracy of >92%. Accuracy was independent of tumor grade (G1, G2, or G3), imaging type (anatomical or functional), or site of tumor (GEP, lung, CUP). For serial follow-up, decreases in NETest2.0^®^ scores were concordant with imaging-based clinical outcomes such as disease stability and treatment response to multiple different therapies. Conversely, increases in scores were concordant with disease recurrence and progression in surgical and in both watch-and-wait and treated cohorts. Our data suggests that a threshold of >0% could provide a sensitive metric for the detection of progression. Such a strategy would allow NETest2.0^®^ to serve as a molecular triage tool, personalizing monitoring while reducing unnecessary radiologic assessments. While a >0% increase provides sensitive detection of molecular activity, a larger increase in sequential NETest2.0^®^ scores (≥+5 to +20%) demonstrated higher specificity for progression in individual cohorts, supporting a tiered risk-stratification approach. Serial NETest2.0^®^ measurements demonstrated concordance with real-world clinical decisions, including initiation of imaging, treatment modification, and assessment of therapeutic efficacy, supporting its clinical role as a surveillance biomarker.

This biomarker-guided approach reflects established practices in other malignancies where circulating tumor biomarkers inform surveillance and clinical decisions. In colorectal cancer, ctDNA-based monitoring predicts recurrence and detects minimal residual disease before imaging can reveal it [24,25]. In melanoma, ctDNA levels correlate with metabolic tumor burden and inform real-time disease status [26]. In localized lung cancer, ctDNA profiling allows early detection of molecular residual disease, frequently preceding radiologic relapse [27]. In advanced lung cancer, ctDNA-guided therapy improves survival by enabling early, targeted treatment adjustments [28]. In pediatric acute lymphoblastic leukemia (ALL), MRD monitoring using quantitative PCR has become standard practice, reducing invasive procedures and improving risk stratification [29]. These examples align with expert consensus from the American Society for Clinical Oncology (ASCO) and the College of American Pathologists (CAP), which supports the clinical use of circulating biomarkers to guide decision-making and reduce unnecessary interventions while maintaining patient safety [30].

For NETs, surgical follow-up is variable based on the range of recommendations in the National Comprehensive Cancer Network (NCCN) guidelines and findings from literature reviews [31]. There is currently no widely accepted postoperative strategy for patient stratification to specify the precise imaging interval within the recommended range [32]. Our study demonstrates that the postoperative use of the NETest2.0^®^ can be used to monitor patients. In the surgical cohorts, patients underwent curative-intent resection with known pathological outcomes. NETest2.0^®^ scores decreased and were normalized in 95% of R0 patients. An elevated post-surgical score was associated with the subsequent development of macroscopic/image detectable disease. This finding is supported by observations in a second surgical cohort (Cohort II), which identified that positive NETest2.0^®^ scores, post-surgery, were associated with the development of detectable disease. The sensitivity, specificity, and accuracy of the NETest2.0^®^ for detecting/predicting disease were all >85%, consistent with an earlier study [33].

For post-operative monitoring following R0 resection, we recommend a binary NETest2.0^®^ readout with a cutoff of 50. Such a binary model produced optimal performance in surgery cohort I, including a sensitivity, specificity, and overall accuracy of 100%. Such an interpretive framework is consistent with other molecular oncology tools, such as ctDNA and MRD assays, where the presence or absence of a detectable molecular signal post-surgery better reflects clinical status than the magnitude of percent change alone. The use of the NETest2.0^®^ to guide post-operative evaluation within the recommended range of imaging intervals for NET patients following surgery (for example, the conversion of a negative to a positive score, as in Surgery Cohort II) has significant potential health economic implications, as it may reduce radiation exposure and lower the costs of diagnostic isotopic imaging [19,34].

We also evaluated the NETest2.0^®^ in both the watch-and-wait and treatment cohorts (Cohorts III and IV). Serial changes in NETest2.0^®^ were closely correlated with disease status: minimal changes were associated with stable disease, while increases (≥+20%) indicated disease progression. This demonstrates that NETest2.0^®^ may detect disease relapses in patients under surveillance. Changes in NETest2.0^®^ scores also accurately correlated with treatment response. Normalization or reduction in scores was linked to clinical response and disease stabilization, as confirmed by imaging. In contrast, score increases were associated with clinical and/or radiologic progression, despite ongoing therapy. These findings were consistent across several different treatment modalities, including SSAs, PRRT, Everolimus, chemotherapy, and CAPTEM, albeit some with smaller numbers (5–7 patients). These observations align with earlier studies in assessing SSA [35,36] and PRRT response, showing stabilization or decrease in follow-up NETest2.0^®^ scores in responders/increase, and increases in non-responders [37,38]. It is reasonable to suggest that effective therapy reduces circulating mRNA expression, which underlies the observed decrease in NETest2.0^®^ scores.

Chromogranin A is an alternative. However, its sensitivity is low, intra- and inter-subject variability is high, and there are multiple causes of false positives [39,40]. Recently, a CgA immunofluorescence assay (KRYPTOR) was evaluated to determine whether it could serve as a surveillance biomarker for predicting disease progression during therapy [23]. While it demonstrates high specificity (93.4%) for detecting “stable or treatment-responsive disease”, its sensitivity for identifying progression or treatment failure was as low as 34.4%, significantly limiting its utility for NET patient monitoring. NETest2.0^®^, in contrast (using the same scoring approach), yielded accuracies (in the 217-patient treatment cohort) of 82.0–84.8% (depending on threshold) with sensitivities of 34.6–72.3% and specificities of 86.4–98.2%. CgA is included as historical context; however, due to known limitations, it is not considered a primary comparator in this study.

Although liquid biopsy has gained traction over the past two decades as a less invasive alternative to tumor biopsy, most clinical research has focused on circulating free DNA (cfDNA) and, to a much lesser extent, circulating tumor cells (CTC). NETs typically do not exhibit a high mutation burden and release few CTCs. Consequently, neither CTCs nor cfDNA have demonstrated clinical utility in the context of NETs [41], and none are currently FDA approved for this indication.

This study should be interpreted in the context of several considerations. First, the analysis is derived from a real-world clinical cohort. While this introduces variability in imaging modality, timing, and follow-up intervals, it reflects routine clinical practice and enhances the generalizability of the findings to intended use settings. Second, imaging was used as the clinical reference standard for disease status and progression. This is consistent with current clinical practice and regulatory precedent, where imaging forms the basis of surveillance and treatment decision-making. The objective of the present study was not to replace imaging, but to evaluate NETest2.0^®^ within this established framework of disease surveillance. Importantly, NETest2.0^®^ is not intended to substitute for imaging in defining the anatomical site or extent of disease progression, nor does it replace histopathological assessment when tissue characterization (e.g., Ki-67 index or tumor heterogeneity) is required. Similarly, clinical decision-making in neuroendocrine tumors remains multidisciplinary, integrating imaging, pathology, and clinical parameters to guide individualized management strategies [42,43]. Third, the duration and frequency of follow-up were variable and, in some cases, relatively limited given the typically indolent course of neuroendocrine tumors. Longer-term follow-up will be valuable to further define the relationship between molecular changes and clinical outcomes. Fourth, while the cohort includes a broad spectrum of clinical states, supporting general applicability, performance may vary across specific subgroups, which may benefit from further context-specific characterization. Finally, economic considerations may influence the routine implementation of molecular surveillance strategies. Formal health-economic evaluations will be important to define the cost-effectiveness of NETest2.0^®^ in optimizing longitudinal disease monitoring.

These considerations support the positioning of NETest2.0^®^ as a complementary, biology-based monitoring tool within an integrated surveillance strategy. Early detection of progression in patients with NETs may avoid ineffective treatment and help guide treatment adaptation. Dedicated multidisciplinary team (MDT) discussion in expert centers represents the gold standard for the management of these patients [42,43]. They have significant value in deciding initial therapeutic strategies in high-grade neuroendocrine patients and are associated with improved overall survival [43] and also with significant management changes in over 60% of NET [42]. We propose that evaluating early molecular changes in NETs could enable more precise treatment adjustments. This would include avoiding unnecessarily prolonged regimens, reducing exposure to adverse events, and limiting the high costs associated with targeted therapies. Tumor progression is a dynamic and sequential process that current methods often fail to capture effectively.

NETest2.0^®^ offers a robust solution for patient monitoring in the post-surgical setting, during watch-and-wait programs, or during active treatment. While a blood-based assay cannot identify the location of new lesions (which can impact MDT decision-making), NETest2.0^®^ fits this paradigm as a validated, peripheral blood-based multi-gene transcriptomic assay. Based on the present findings, NETest 2.0 has the potential to:

- Identify molecular changes that may precede radiologic progression.
- Support risk-adapted surveillance strategies based on molecular stability.
- Improve patient experience by potentially reducing unnecessary imaging in molecularly stable patients.
- Enable more efficient resource utilization within biomarker-guided care pathways.

This approach is consistent with monitoring paradigms in other cancers [24,26,27,29,30]. NETest2.0^®^ is not intended to replace imaging. It complements imaging by enabling biology-guided surveillance, potentially reducing unnecessary imaging frequency in stable patients while supporting earlier imaging in molecular progressors [15]. Inclusion of NETest2.0^®^ in MDT discussions could amplify the success of patient management protocols.

## 5. Conclusions

NETest2.0^®^ has clinical value in several commonly encountered clinical scenarios involving NET patients. It can be used for post-surgical assessment (is there MRD?), warn of potential post-operative disease prior to image-detectable recurrence in “cured” patients, be useful in the detection of progression in watch-and-wait scenarios, and, finally, help determine whether a treatment may be efficacious. For these individual situations, we identified that this enables a risk-adapted surveillance model: patients demonstrating molecular stability or decline (≤−5%) may defer imaging, while others could proceed with confirmatory scans. We acknowledge that different clinical situations could use different thresholds. Evaluation of NETest2.0^®^ performance across multiple Δ thresholds in the 404-patient cohort, with the trade-offs between sensitivity and specificity, provides flexibility to the treating physician to personalize patient management across different clinical contexts. These data demonstrated that NETest2.0^®^ is effective across all NET grades and all major NET primary sites and is independent of imaging modality and patient management paradigms. Such a strategy allows NETest2.0^®^ to serve as a molecular triage tool, personalizing monitoring while reducing unnecessary radiologic assessments. NETest^®^ 2.0 is not intended to replace imaging. It complements imaging by enabling biology-guided surveillance, potentially reducing unnecessary imaging frequency in stable patients while supporting earlier imaging in molecular progressors.

## Figures and Tables

**Table 1:** Demographics—Objective 2.

	Cohort #1	Cohort #2	Cohort #3	Cohort #4
Number	71	44	72	217
Age	58 (26–82)	55.5 (30–78)	62 (19–86)	62 (29–88)
Gender (M:F)	42:29	21:23	33:39	118:99
Site Stomach Pancreas Small Bowel Appendix Colon/rectum Lung CUP	1 (1.4%) 43 (60.6%) 24 (33.8%) 0 (0%) 1 (1.4%) 2 (2.8%) 0 (0%)	0 (0%) 20 (45.5%) 23 (52.3%) 1 (2.2%) 0 (0%) 0 (0%) 0 (0%)	4 (5.6%) 27 (37.5%) 30 (41.7%) 1 (1.4%) 1 (1.4%) 2 (2.7%) 7 (9.7%)	3 (1.4%) 85 (39.2%) 94 (43.3%) 0 (0%) 6 (2.8%) 15 (6.9%) 14 (6.4%)
Grade G1 G2 G3 ND	47 (66.2%) 23 (32.4%) 1 (1.4%) 0 (0%)	26 (59.1%) 17 (38.6%) 1 (2.3%) 0 (0%)	33 (45.8%) 31 (43.1%) 0 (0%) 8 (11.1%)	74 (34.1%) 115 (53%) 26 (12%) 2 (0.9%)
Lymph node N0 N1	34 (47.9%) 37 (52.1%)	21 (47.7%) 23 (52.3%)	25 (34.7%) 47 (65.3%)	32 (14.7%) 185 (85.3%)
Liver M0 M1	63 (88.7%) 8 (11.3%)	36 (81.8%) 8 (18.2%)	46 (63.9%) 26 (36.1%)	58 (26.7%) 159 (73.3%)

ND = no data.

**Table 2:** NETest2.0^®^ performance characteristics for Objective 2 (*n* = 404).

Metrics	NETest2.0^®^ Score #2 ≥ 50 with Increase in Second Sample from First Sample				
Thresholds of increase in the follow-up sample from initial sample	Any increase (>0%)	>+5%	>+10%	>+15%	>+20%
Sensitivity	78.0% (69–85%)	67.8% (59–76%)	58.5% (49–68%)	51.7% (42–61%)	43.2% (34–53%)
Specificity	83.2% (78–87%)	90.6% (87–94%)	93.7% (90–96%)	97.2% (95–99%)	98.3% (96–99%)
PLR	4.65 (3.53–6.12)	7.18 (4.91–10.50)	9.29 (5.79–14.9)	18.48 (9.13–37.41)	24.72 (10.12–60.39)
NLR	0.26 (0.19–0.37)	0.36 (0.27–0.46)	0.44 (0.36–0.55)	0.50 (0.41–0.60)	0.58 (0.49–0.68)
PPV	65.7% (59–72%)	74.8% (67–81%)	79.3% (71–86%)	88.4% (79–94%)	91.1% (81–96%)
NPV	90.2% (87–93%)	87.2% (84–90%)	84.5% (82–87%)	83.0% (80–86%)	80.8% (78–83%)
Accuracy	81.7% (78–85%)	83.9% (80–87%)	83.4% (79–87%)	83.9% (80–87%)	82.2% (78–86%)

**Table 3:** NETest2.0^®^ performance characteristics for Objective 2—GEP-NET and CUP cohort (*n* = 386).

Metrics	NETest2.0^®^ Score #2 ≥ 50 with Increase in Second Sample from First Sample				
Increase thresholds	Any increase (>0%)	>+5%	>+10%	>+15%	>+20%
Sensitivity	76.3% (67–84%)	69.4% (60–78%)	61.3% (52–70%)	54.1% (44–64%)	45.0% (36–55%)
Specificity	82.9% (78–87%)	90.2% (86–93%)	93.5% (90–96%)	97.1% (94–99%)	98.2% (96–99%)
PLR	4.47 (3.38–5.91)	7.07 (4.84–10.32)	9.36 (5.9–15.0)	18.58 (9.2–37.6)	24.77 (10.2–60.5)
NLR	0.29 (0.20–0.40)	0.34 (0.26–0.45)	0.41 (0.33–0.52)	0.47 (0.39–0.58)	0.56 (0.47–0.66)
PPV	64.9% (58–71%)	74.0% (66–81%)	79.1% (70–86%)	88.2% (79–94%)	90.9% (80–96%)
NPV	89.4% (85–92%)	87.9% (85–91%)	85.7% (83–88%)	84.0% (81–87%)	81.6% (79–84%)
Accuracy	81.0% (77–85%)	84.2% (80–88%)	84.2% (80–87%)	84.7% (81–88%)	82.9% (79–87%)

**Table 4:** NETest2.0^®^ performance characteristics for Objective 2—GEP-NET cohort (*n* = 369).

Metrics	NETest2.0^®^ Score #2 ≥ 50 with Increase in Second Sample from First Sample				
Increase thresholds	Any increase (>0%)	>+5%	>+10%	>+15%	>+20%
Sensitivity	79.0% (70–86%)	69.5% (60–78%)	61.0% (51–70%)	53.3% (43–63%)	44.8% (35–55%)
Specificity	82.2% (77–87%)	89.8% (86–93%)	93.2% (89–96%)	97.0% (94–99%)	98.1% (96–99%)
PLR	4.44 (3.36–5.86)	6.80 (4.65–9.93)	8.94 (5.58–14.32)	17.60 (8.69–35.6)	23.63 (9.67–57.77)
NLR	0.25 (0.18–0.38)	0.34 (0.25–0.45)	0.42 (0.33–0.53)	0.48 (0.39–0.59)	0.56 (0.47–0.67)
PPV	63.9% (57–70%)	73.0% (65–80%)	78.1% (69–85%)	87.5% (78–93%)	90.4% (79–96%)
NPV	90.8% (87–95%)	88.1% (85–91%)	85.7% (83–88%)	83.9% (81–87%)	81.7% (79–84%)
Accuracy	81.3% (77–85%)	84.0% (80–88%)	84.0% (81–88%)	84.6% (81–88%)	82.9% (79–87%)

## Data Availability

Due to privacy and ethical concerns, the data that support the findings of this study are not publicly available but are available on request from the corresponding author.

## Supplementary Materials

The following supporting information can be downloaded at: https://www.mdpi.com/article/10.3390/cancers18091457/s1, Supplement including five figures (sub-analysis of 886 patients, 2 × 2 tables for different cut-offs in the 404, 386, and 369 cohorts, and an evaluation of the impact of disease burden on metrics).

## Abbreviations

The following abbreviations are used in this manuscript:

MRD	Minimal residual disease
RT-PCR	Reverse-transcription polymerase chain reaction
NPV	Negative predictive value
PPV	Positive predictive value
IQR	Interquartile range
CTC	Circulating tumor cell
STARD	Standards for Reporting and Diagnostic Accuracy Studies
CI	Confidence interval
ROC	Receiver operating characteristic
AUC	Area under the curve
SD	Standard deviation
NET	Neuroendocrine tumor
PRRT	Peptide receptor radionuclide therapy
CT	Computed tomography
MRI	Magnetic resonance imaging
SSA	Somatostatin analogue
PET	Positron emission tomography
CgA	Chromogranin A
IVD	In vitro diagnostic
GEP	Gastroenteropancreatic
BP	Bronchopulmonary
CUP	Cancer of unknown primary
IPD	Image-positive disease
IND	Image-negative disease
PD	Progressive disease
SD	Stable disease
PR	Partial responses
