# Supplementary material for: Clinical Validity of NETest2.0® in Surveillance of Neuroendocrine Tumor Patients: Evidence from a NET Registry Study (NCT02270567)
Source: Cancers (Basel). 2026 May 1;18(9):1457. doi: 10.3390/cancers18091457 (PMC13163055; doi:10.3390/cancers18091457)
Supplement: Supplementary file 1 [file cancers-18-01457-s001.zip › cancers-4273958-supplementary.pdf]

Supplementary Materials

Clinical Validity of NETest2.0® in Surveillance of Neuroendocrine Tumor Patients: Evidence from a NET Registry Study (NCT02270567)

Anthony Gulati [1], Diane Reidy [2], Abdel Halim [3,*], Kiarash Mashayekhi [4], David K. Imagawa [5] and Daniel M. Halperin [6]

1 Department of Medical Oncology, Stamford Health, Stamford, CT 06902, USA; agulati@stamhealth.org
2 Department of Medical Oncology, Duke Cancer Institute, Durham, NC 27110, USA; diane.reidy-lagunes@duke.edu
3 Wren Laboratories, Branford, CT 06405, USA
4 Department of Surgery, University of North Dakota School of Medicine & Health Sciences, Grand Forks, ND 58203, USA; kmashay1@hs.uci.edu
5 Department of Surgery, University of California, Irvine Medical Center, Irvine, CA 92868, USA; dkimagaw@hs.uci.edu
6 Department of Hematology and Medical Oncology, Emory University, Atlanta, GA 30322, USA; daniel.mark.halperin@emory.edu
* Correspondence: ahalim@wrenlaboratories.com; Tel.: +1-203-208-3464

Supplementary Figures

	Imaging positive	Imaging negative	Total
NETest positive	695	37	732
NETest negative	26	128	154
Total	721	165	886

Statistic	Value	95% CI
Sensitivity	96.39%	94.76% to 97.63%
Specificity	77.58%	70.44% to 83.69%
PLR	4.3	3.24 to 5.71
NLR	0.05	0.03 to 0.07
PPV	94.95%	93.39% to 96.15%
NPV	83.12%	76.99% to 87.87%
Accuracy	92.89%	90.99% to 94.49%

A

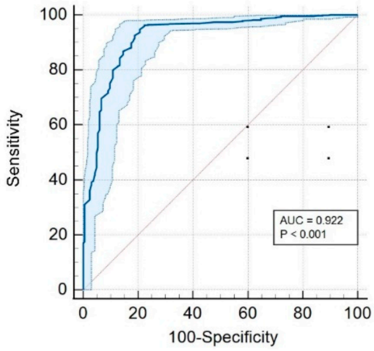

B

Figure S1. Assessment of NETest2.0® (positive/negative) for detecting NET disease in 886 individual patients. **S1A**. A 2x2 table and the corresponding diagnostic metrics for detecting disease. **S1B**. AUROC for disease detection including the z-statics (32.81) and significance (*p*<0.0001). Abbreviations: AUROC: Area under the Receiver Operator Curve, AUC: Area under the Curve; NLR = negative likelihood ratio, NPV = negative predictive values, PLR = positive likelihood ratio, PPV = positive predictive value. NLR = negative likelihood ratio, NPV = negative predictive values, PLR = positive likelihood ratio, PPV = positive predictive value.

0	Progressive	Stable	Total
Threshold >0	92	48	140
Threshold ≤0	26	238	264
Total	118	286	**404**

5	Progressive	Stable	Total
Threshold >5	80	27	107
Threshold ≤5	38	259	297
Total	118	286	404

10	Progressive	Stable	Total
Threshold >10	69	18	87
Threshold ≤10	49	268	317
Total	118	286	404

15	Progressive	Stable	Total
Threshold >15	61	8	69
Threshold ≤15	57	278	335
Total	118	286	404

20	Progressive	Stable	Total
Threshold >20	51	5	56
Threshold ≤20	67	281	348
Total	118	286	404

Figure S2. 2x2 tables for thresholds 0 to 20 for the 404 cohort. This approach is based on the FDA-approved approach for Chromogranin A per the CASPAR study [1]. The following Δ thresholds (change between 2nd and 1st score): >0%, >+5%, >+10%, >+15%, >+20%, were evaluated in the entire 404 cohort.

**0**	**Progressive**	**Stable**	**Total**
Threshold >0	87	47	134
Threshold ≤0	24	228	252
Total	111	275	**386**

**5**	**Progressive**	**Stable**	**Total**
Threshold >5	77	27	104
Threshold ≤5	34	248	282
Total	111	275	386

**10**	**Progressive**	**Stable**	**Total**
Threshold >10	68	18	86
Threshold ≤10	43	257	300
Total	111	275	386

**15**	**Progressive**	**Stable**	**Total**
Threshold >15	60	8	68
Threshold ≤15	51	267	318
Total	111	275	386

**20**	**Progressive**	**Stable**	**Total**
Threshold >20	50	5	55
Threshold ≤20	61	270	331
Total	111	275	386

Figure S3. 2x2 tables for thresholds 0 to 20 for the 386 cohort. This approach is based on the FDA-approved approach for Chromogranin A per the CASPAR study [1]. The following Δ thresholds (change between 2nd and 1st score): >0%, >+5%, >+10%, >+15%, >+20%, were evaluated in the entire 386 cohort.

0	Progressive	Stable	Total
Threshold >0	83	47	130
Threshold ≤0	22	217	239
Total	105	264	**369**

5	Progressive	Stable	Total
Threshold >5	73	27	100
Threshold ≤5	32	237	269
Total	105	264	369

10	Progressive	Stable	Total
Threshold >10	64	18	82
Threshold ≤10	41	246	287
Total	105	264	369

15	Progressive	Stable	Total
Threshold >15	56	8	64
Threshold ≤15	49	256	305
Total	105	264	369

20	Progressive	Stable	Total
Threshold >20	47	5	52
Threshold ≤20	58	259	317
Total	105	264	369

Figure S4. 2x2 tables for thresholds 0 to 20 for the 369 cohort. This approach is based on the FDA-approved approach for Chromogranin A per the CASPAR study [1]. The following Δ thresholds (change between 2nd and 1st score): >0%, >+5%, >+10%, >+15%, >+20%, were evaluated in the entire 369 GEP-NET cohort.

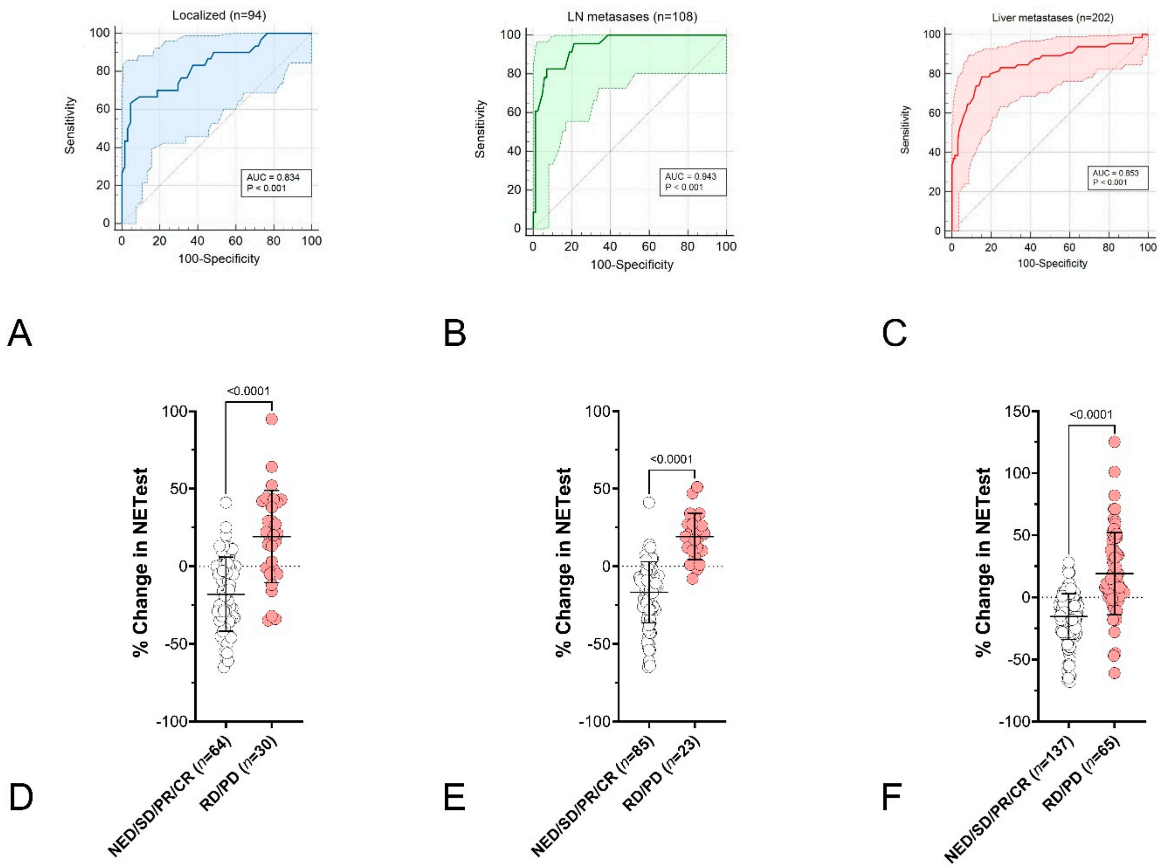

Figure S5. AUROC analyses and % changes based on disease burden. AUROC for disease detection in 94 localized. NETs (**S5A**), for 108 patients with lymph node (LN) metastases (**S5B**) and for 202 patients with liver metastases (**S5C**). % changes in patients with localized disease (**S5D**), LN disease (**S5E**) and liver disease (**S5F**). Changes were significantly different between NED/SD/responders vs. Residual disease/non-responders. *% Change = Mean±SD. NED = no evidence of disease, PR = partial responder, CR = complete responder, SD = stable disease, RD = residual disease, PD = progressive disease.*

References

1. Meng, Q.H.; Halfdanarson, T.R.; Bornhorst, J.A.; Jann, H.; Shaheen, S.; Shi, R.Z.; Schwabe, A.; Stade, K.; Halperin, D.M. Circulating Chromogranin A as a Surveillance Biomarker in Patients with Carcinoids-The CASPAR Study. *Clin. Cancer Res.* **2024**, *30*, 5559–5567. https://doi.org/10.1158/1078-0432.ccr-24-1875.
